# Development of a Cold-Adapted *Pseudoalteromonas* Expression System for the *Pseudoalteromonas* Proteins Intractable for the *Escherichia coli* System

**DOI:** 10.1371/journal.pone.0137384

**Published:** 2015-09-02

**Authors:** Zi-Chao Yu, Bai-Lu Tang, Dian-Li Zhao, Xiuhua Pang, Qi-Long Qin, Bai-Cheng Zhou, Xi-Ying Zhang, Xiu-Lan Chen, Yu-Zhong Zhang

**Affiliations:** 1 State Key Laboratory of Microbial Technology, Shandong University, Jinan, China; 2 Marine Biotechnology Research Center, Shandong University, Jinan, China; Weizmann Institute of Science, ISRAEL

## Abstract

Although the *Escherichia coli* expression system is the most commonly used expression system, some proteins are still difficult to be expressed by this system, such as proteins with high thermolability and enzymes that cannot mature by autoprocessing. Therefore, it is necessary to develop alternative expression systems. In this study, a cold-adapted *Pseudoalteromonas* expression system was developed. A shuttle vector was constructed, and a conjugational transfer system between *E*. *coli* and psychrophilic strain *Pseudoalteromonas* sp. SM20429 was established. Based on the shuttle vector, three reporter vectors were constructed to compare the strength of the cloned promoters at low temperature. The promoter of xylanase gene from *Pseudoalteromonas* sp. BSi20429 was chosen due to its high activity at 10–15°C. An expression vector pEV containing the chosen promoter, multiple cloning sites and a His tag was constructed for protein expression and purification. With pEV as expression vector and SM20429 as the host, a cold-adapted protease, pseudoalterin, which cannot be maturely expressed in *E*. *coli*, was successfully expressed as an active extracellular enzyme when induced by 2% oat spelt xylan at 15°C for 48 h. Recombinant pseudoalterin purified from the culture by Ni affinity chromatography had identical N-terminal sequence, similar molecular mass and substrate specificity as the native pseudoalterin. In addition, another two cold-adapted enzymes were also successfully expressed by this system. Our results indicate that this cold-adapted *Pseudoalteromonas* expression system will provide an alternative choice for protein expression, especially for the *Pseudoalteromonas* proteins intractable for the *E*. *coli* system.

## Introduction

The *Escherichia coli* expression system is the most commonly used system for expressing recombinant proteins. Innumerable proteins have been successfully expressed by this system [1, 2]. However, the *E*. *coli* expression system is not suitable for all proteins. Some proteins that cannot undergo autoprocessing are difficult to be expressed in this system due to the lack of proteases for protein processing. In addition, *E*. *coli* is a mesophilic bacterium; optimal growth occurs at 37°C. Some cold-adapted proteins are not expressed well in *E*. *coli* at 37°C due to their low thermostability. Although reducing the induction temperature may improve the expression of some cold-adapted proteins [1], it is not always effective and the expression time can be prolonged at a low temperature. For these reasons, it is necessary to develop alternative expression systems to produce proteins that are difficult to express via the *E*. *coli* expression system.

*Pseudoalteromonas* is widespread throughout the sea, including cold environments, such as deep-sea and polar regions, from which a large number of psychrophilic *Pseudoalteromonas* species have been isolated [3, 4]. Many of these psychrophilic *Pseudoalteromonas* species have the ability to produce large amount of various extracellular cold-adapted enzymes and exopolysaccharides [3, 5–9]. However, some enzymes from *Pseudoalteromonas* cannot be maturely expressed in the *E*. *coli* expression system [10, 11], which impedes the further study of these enzymes. By far, a cold-adapted expression system has been developed in *Pseudoalteromonas haloplanktis* TAC125 [12, 13], indicating that some cold–adapted *Pseudoalteromonas* strains can be used to develop cold-adapted expression system.

Pseudoalterin, which is secreted by *Pseudoalteromonas* sp. CF6-2 from deep-sea sediment, is a novel metalloprotease that belongs to the M23A subfamily [11]. It can efficiently hydrolyze elastin using a novel elastolytic mechanism that is different from that of other M23 metalloproteases [11]. Because elastin is abundant in marine animals, it represents a significant amount of organic nitrogen. Pseudoalterin and other M23 metalloproteases secreted by many marine bacteria may play an important role in elastin degradation and nitrogen recycling [11]. The optimal temperature for pseudoalterin is 25°C. At 35°C, its half-life is 14 min, indicating that pseudoalterin is a cold-adapted enzyme that is highly thermolabile [11]. Pseudoalterin and other M23 proteases cannot be expressed in their mature form via autoprocessing because the cleavage of their propeptides requires the help of other enzymes [14]. For this reason, pseudoalterin is unable to be expressed in its mature form in *E*. *coli* [11], which severely impedes the further study of its structure and function.

The cryptic plasmid pSM429 was previously isolated from the psychrophilic *Pseudoalteromonas* sp. BSi20429 (CCTCC Accession No. M2010239,hereafter called BSi20429), which was isolated from the Arctic sea ice [15]. The minimal replicon of pSM429 was determined by constructing the shuttle vector pWD and its derivatives and developing an electroporation system. Using the pWD2 vector and driven by the *Plac* promoter from mesophilic *E*. *coli*, the erythromycin resistance gene and the gene encoding the catalytic domain of cold-adapted cellulase Cel308 were successfully expressed in *Pseudoalteromonas* sp. SM20429, a plasmid-cured strain of *Pseudoalteromonas* sp. BSi20429 [15]. However, because the *Plac* promoter is from a mesophilic organism, proteins cannot be expressed with vector pWD2 at temperatures lower than 25°C, even though SM20429 is a psychrophilic bacterium [15]. In this study, we further improved this cold-adapted heterologous expression system. Interspecies conjugation was performed to obtain a high transfer frequency. To screen for a strong promoter at a low temperature, the strength of the promoters for a cold shock protein gene, a heat shock protein gene and an xylanase gene from BSi20429 were compared. Based on the results, an expression vector, pEV, was constructed, which contained the promoter of the xylanase gene, multiple cloning sites, and a His tag. Using vector pEV, pseudoalterin and other two cold-adapted proteins were successfully expressed in SM20429 at 15°C and purified by Ni affinity chromatography. More importantly, although pseudoalterin cannot be maturely expressed in *E*. *coli*, it was expressed as an active enzyme in SM20429. Our results support the potential use of this expression system for unautoprocessed *Pseudoalteromonas* proteins and cold-adapted proteins that are intractable for the *E*. *coli* expression system.

## Materials and Methods

### Plasmids, bacterial strains and growth conditions

The plasmids and bacterial strains that were used in this study are described in Table 1. *Pseudoalteromonas* sp. SM20429 (hereafter called SM20429) was grown at 15–20°C in a marine LB broth (10 g of peptone, 5 g of yeast extract, 1 l of artificial seawater, pH 7.5). *E*. *coli* DH5α was used as the host for gene cloning and was grown at 37°C in LB medium [16]. *E*. *coli* ET12567 [17] harboring the plasmid pUZ8002 [18] was used as the donor in intergeneric conjugation experiments and was cultured at 37°C in LB medium containing chloromycetin (25 μg/ml) and kanamycin (100 μg/ml).

**Table 1:** Strains and plasmids used in this study.

Strains or plasmids	Genotype or markers; characteristics and uses	Source or reference
Strains		
*Escherichia coli* DH5α	*supE* 44, △*lac*U169 (θ80*lacZ*△*M15*) *endA1*, *recA1*, *hsdR17*, *thi-1* l− *gyrA96*, *relA1*; gene cloning	Transgen, China
*Escherichia coli* ET12567	*dam*, *dcm*, *hsdM*, *hsdS*, *hsdR*, *cat*, *tet*; donor strain for conjugal transfer	[17]
*Pseudoalteromonas* sp. BSi20429	Wild type	This work
*Pseudoalteromonas* sp. SM20429	Plasmid cured strain of *Pseudoalteromonas* sp. BSi20429; host for heterologous expression	[15]
*Pseudoalteromonas* sp. SM9913	Wild type	This work
*Pseudoalteromonas* sp. Bsw20308	Wild type	This work
plasmids		
pUZ8002	*tra*, *neo*, RP4	[18]
pKNG101	R6K-derived suicide plasmid containing Str and sacB; template for *oriT* and *sacB* amplification	[19]
pWD2	Shuttle vector that constructed previously; template for *RepA* and *CAT* amplification	[15]

### Construction of the shuttle vector pOriT-4CM and conjugal transfer

A shuttle vector for conjugational transfer was constructed based on pGEM-T easy vector and the plasmid pWD2 [15]. Gene *oriT*, the origin of transfer, was amplified from pKNG101 [19] with primers OriTF/OriTR (Table 2) and was cloned into a pGEM-T easy vector (Promega, Wisconsin, USA). The derivative vector was named pV1. A DNA fragment containing the chloramphenicol acetyl transferase (CAT) cassette and the gene *repA* as well as its flanking sequences was amplified from plasmid pWD2 [15] using primers ZF/CmR and was cloned into the *Pst*I and *Spe*I sites of pV1. The derivative vector was named pOriT-4CM. Gene *repA* and its flanking sequences are responsible for the stable segregation of pOriT-4CM in SM20429. The CAT cassette was used as a selective marker. The plasmid pOriT-4CM was sequenced by Biosune Inc. (China).

**Table 2:** Primers used in this study.

Primer	Sequence (5’-3’)
OriTF	GCCAGCTCGTCGGTGTAGC
OriTR	CAACAACGTTGCCCGGATCG
ZF	TGCACTGCAGCAAGACACTGTGAAGGC
CmR	GGACTAGTGAAGCACACGGTCACACTG
XlyF	ACATGCATGCGGCGAAAAATGCGCTAATTAACAG
XlyR	GTGATTAATTAAACTGTTATCCATGATGTACCTGTTGTGTG
CenXF	CACACAACAGGTACATCATGGATAACAGTTTAATTAATCAC
CenR	ATAAGAATGCGGCCGCCTATTAGCCACCCCAACCTTGAATG
CspAF	ACATGCATGCGTTTCATCACTGAGATAAGCC
CspAR	GTGATTAATTAAACTGTTATCCATAAGAGCGGGTGTAGC
CenCF	GCTACACCCGCTCTTATGGATAACAGTTTAATTAATCAC
dnaKF	ACATGCATGCGCATGAAGATGTCCTTTTATAGTGC
dnaKR	GTGATTAATTAAACTGTTATCCATGAAACTTCTCCAACTTC
CendF	GAAGTTGGAGAAGTTTCATGGATAACAGTTTAATTAATCAC
1266F	GGAAGATCTATGGCTATTAAAGTATTAAG
1266R	CCGCTCGAGATCTTTAAAGTGTTGCTTAATTTTAGC
1267F	GGAAGATCTATGACAATTAAAACAGTTTCAGTTG
1267R	CCGCTCGAGCAAAATACCGCGAGTATCAATAAC
PsnF	GGAAGATCTATGAATAAACATTTACTAACACTTGC
PsnR	CCGCTCGAGACGGTATAAGGGTCGCCAAG
PsnEF	CGCGCGGATCCATGAATAAACATTTACTAACACTTGC
PsnER	CGCGCGGAATCCACGGTATAAGGGTCGCCAAG
UEF	CGCCATATGATGGCTATTAAAGTATTAAG
UER	CCGCTCGAGATCTTTAAAGTGTTGCTTAATTTTAGC
UDF	CGCCATATGATGACAATTAAAACAGTTTCAGTTG
UDR	CCGCTCGAGCAAAATACCGCGAGTATCAATAAC

The sequences underlined represent the restriction sites.

The shuttle vector pOriT-4CM was transferred into SM20429 by conjugation with *E*. *coli* ET12567 (pUZ8002) cells harboring pOriT-4CM as the donor. The donor cells and the SM20429 cells were both grown to the logarithmic phase (OD_600_ ≈ 0.6) and combined at a 100:1 ratio after washing them twice using LB for the donor cells and marine LB for the SM20429 cells. The mixture was spread onto sterile 0.45 μm-pore-size membranes that were laid on marine LB plates. After 24 h of mating at 20°C, the cells were resuspended in 1 ml of marine LB medium and were spread onto marine LB plates containing 50 μg/ml ampicillin and 12.5 μg/ml chloramphenicol. The plates were then incubated at 20°C. Because the donor *E*. *coli* ET12567 could not survive on the selective plate at 20°C after mating with *Pseudoalteromonas* stains for an unknown reason [20], all the coloies grown on the plates were transconjugant colonies. The transconjugants were then cultured at 20°C for 24 h in 5 ml of marine LB broth containing 50 μg/ml ampicillin and 12.5 μg/ml chloramphenicol. Plasmids were extracted from the transconjugants using the High-purity Plasmid DNA Mini-preparation Kit (Bioteke, Beijing, China).

### Construction of the promoter reporter vectors

The genome sequence of BSi20429 has been deposited in databases under the DDBJ/GenBank/EMBL accession number BADV00000000. The promoter reporter vectors were constructed using the putative promoters of the xylanase gene (GenBank Accession No. GAA68803.1), the cold shock protein gene *cspA* (GenBank Accession No. GAA67962.1) and the heat shock protein gene *dnaK* (GenBank Accession No. GAA68962.1) from BSi20429. A DNA fragment from the Arctic sea ice strain *Pseudoalteromonas* sp. BSw20308 that encodes the catalytic domain and the signal peptide of the cold-adapted cellulase Cel308 (GenBank Accession No. HQ997897) was used as the reporter gene.

Based on the genome sequence of BSi20429, the upstream region (200 bp) of the xylanase gene containing the gene promoter was amplified from SM20429 with primers XlyF/XlyR. The reporter gene was amplified from the genomic DNA of *Pseudoalteromonas* sp. BSw20308 (CCTCC Accession No. AB2015018) with primers CenXF/CenR. Because the primers XlyR and CenXF are complete reverse complements, the two DNA fragments were spliced by overlap PCR with the *Sph*I site at the 5’ end and the *Not*I site at the 3’ end. The resultant DNA fragment containing the xylanase gene promoter and the reporter gene was then cloned into the *Sph*I and *Not*I sites of pOriT-4CM. The constructed promoter reporter vector was named p4xc. The other two promoter reporter vectors, p4cc and p4dc, were constructed using the same method as p4xc. p4cc contained the promoter of *cspA*, p4dc contained the promoter of *dnaK*, and both contained the same reporter gene as p4xc. All the sequences of the promoter regions in these vectors were verified by sequencing.

### Cellulase assay of the expressed reporter gene

The chosen reporter vector (p4xc, p4cc or p4dc) was transferred into SM20429 by conjugation. SM20429 harboring the reporter vector was grown in 100 ml marine LB broth containing 50 μg/ml ampicillin and 12.5 μg/ml chloramphenicol at different temperatures (5°C, 10°C, 15°C) with shaking, and the extracellular cellulase activity was measured at different culture times. Samples collected at different culture times were centrifuged (13,000 rpm, 1 min) at 4°C, and the cellulase activity in the supernatant was measured as previously described [21]. A mixture containing 100 μl of the supernatant and 900 μl of 1% sodium carboxyl methyl cellulose was incubated at 35°C for 30 min. After incubation, the amount of released reducing sugar was measured using the dinitrosalicylic acid (DNS) method and glucose was used as a standard [22]. The experiments were repeated three times. Three parallels were set in each assay, and errors were calculated.

### Construction of the expression vectors

A DNA fragment containing the promoter of the xylanase gene, multiple cloning sites and a His tag (a sequence of 6 repeated CACs) was synthesized, in which the *Sph*I site was introduced at the 5’ end and the *Not*I site at the 3’ end. The DNA fragment was cloned into the *Sph*I and *Not*I sites of pOriT-4CM, and an expression vector was constructed, called pEV. Several genes encoding cold-adapted proteins from deep sea psychrophilic *Pseudoalteromonas* strains were used as target genes for protein expression and purification. From strain *Pseudoalteromonas* sp. SM9913 (CCTCC Accession No. M2010336), genes that encode UDP-GlcNAc 2-epimerase (GenBank Accession No. ADT68205.1) and UDP-ManNAc dehydrogenase (GenBank Accession No. ADT68206.1) were used. The gene (GenBank Accession No. HQ005379.1) that encodes pseudoalterin from strain *Pseudoalteromonas* sp. CF6-2 was also included. These three genes were amplified by PCR using the following primers: 1266F/1266R for the UDP-GlcNAc 2-epimerase gene, 1267F/1267R for the UDP-ManNAc dehydrogenase gene and PsnF/PsnR for the pseudoalterin gene. Then, they were cloned into the pEV vector at the *Bgl*II and *Xho*I sites. The constructed expression vectors were named pEV-UDP-E (UDP-GlcNAc 2-epimerase), pEV-UDP-D (UDP-ManNAc dehydrogenase) and pEV-Psn (pseudoalterin).

In addition, three expression vectors were also constructed to express these proteins in the *E*. *coli* system. The gene encoding pseudoalterin was cloned into the pGEX-4T-1 vector (GE, Little Chalfont, UK) containing a GST tag. The gene encoding UDP-GlcNAc 2-epimerase and the gene encoding UDP-ManNAc dehydrogenase were also cloned separately into a pET-28a-c (+) vector (Novagen, Wisconsin, USA) containing a His tag.

### Protein expression

The constructed vector pEV-Psn was transferred into SM20429 by conjugation and the conditions for pseudoalterin expression were optimized. SM20429 harboring pEV-Psn was cultured to logarithmic phase (OD_600_≈0.6) at 20°C in 50 ml marine LB broth containing 50 μg/ml ampicillin and 12.5 μg/ml chloramphenicol. Different concentrations of oat spelt xylan (1%, 2% and 3%) were added in the culture prior to incubation at 15°C with shaking at 100 rpm. Samples were collected at different culture times and the extracellular elastolytic activity was measured as previously described [11] to determine the optimal xylan concentration for pseudoalterin expression. To determine the optimal culture temperature, cultures with 2% oat xylan were grown at 5°C, 10°C, 15°C or 20°C, and the extracellular elastolytic activity was measured at different culture times. The experiments were repeated three times. Three parallels were set in each assay, and errors were calculated. UDP-GlcNAc 2-epimerase and UDP-ManNAc dehydrogenase were expressed in SM20429 under the same optimal conditions as for pseudoalterin expression. SM20429 harboring pEV-UDP-E or pEV-UDP-D was cultured to logarithmic phase (OD_600_≈0.6) at 20°C and then induced by 2% oat spelt xylan at 15°C for 48 h.

To express these proteins in *E*. *coli*, the three constructed expression vectors were transformed into *E*. *coli* BL21 (DE3) separately. *E*. *coli* BL21 (DE3) harboring an expression vector was cultured to logarithmic phase (OD_600_≈0.6–0.8) at 37°C and then induced by IPTG at 15°C.

### Protein purification

To purify the proteins fused with the His tag, cultures of 300 ml were first centrifuged for 10 min at 10,000 rpm. When the recombinant protein was an extracellular protein, the supernatant was concentrated with PEG20000 prior to dialysis in Binding buffer (50 Mm Tris-Cl pH 8.0, 200 mM NaCl, 5 mM imidazole, 1% glycerol). When the recombinant protein was an intracellular protein, the precipitate was resuspended in Binding buffer, sonicated in an ice-water bath and centrifuged for 10 min at 10,000 rpm. Then, the recombinant proteins were purified using Ni Sepharose 6 Fast Flow (GE, Little Chalfont, UK) according to the manufacturer’s instructions.

To purify the recombinant pseudoalterin fused with the GST tag, the culture of *E*. *coli* BL21 (DE3) harboring the expression vector was centrifuged for 5 min at 10,000 rpm. The precipitate was resuspended in 50 mM phosphate buffer (pH 7.2) containing 1 mM DTT, sonicated in an ice-water bath and centrifuged for 10 min at 10,000 rpm. From the solution, the recombinant pseudoalterin was purified with Glutathione Sepharose ^TM^ 4 Fast Flow (GE, Little Chalfont, UK) according to the manufacturer’s instructions.

### N-terminal sequence analysis of the recombinant pseudoalterin

The purified recombinant pseudoalterin was electrophoresed using an SDS-polyacrylamide gel and transferred to a PVDF transfer membrane (GE, Little Chalfont, UK). The N-terminal amino acid sequence was analyzed by Edman degradation with PROCISE491 (Applied Biosystems, Foster City, CA) at Beijing University (Beijing, China).

### Substrate specificity of recombinant pseudoalterin and protein determination

The activities of recombinant pseudoalterin on casein, elastinorcein, gelatin and collagen were determined as previously described [11]. The protein concentration was determined by Bradford assay [23] using bovine serum albumin (Sigma, USA) as the standard.

## Results

### Construction of the conjugational transfer system

**Fig 1:**
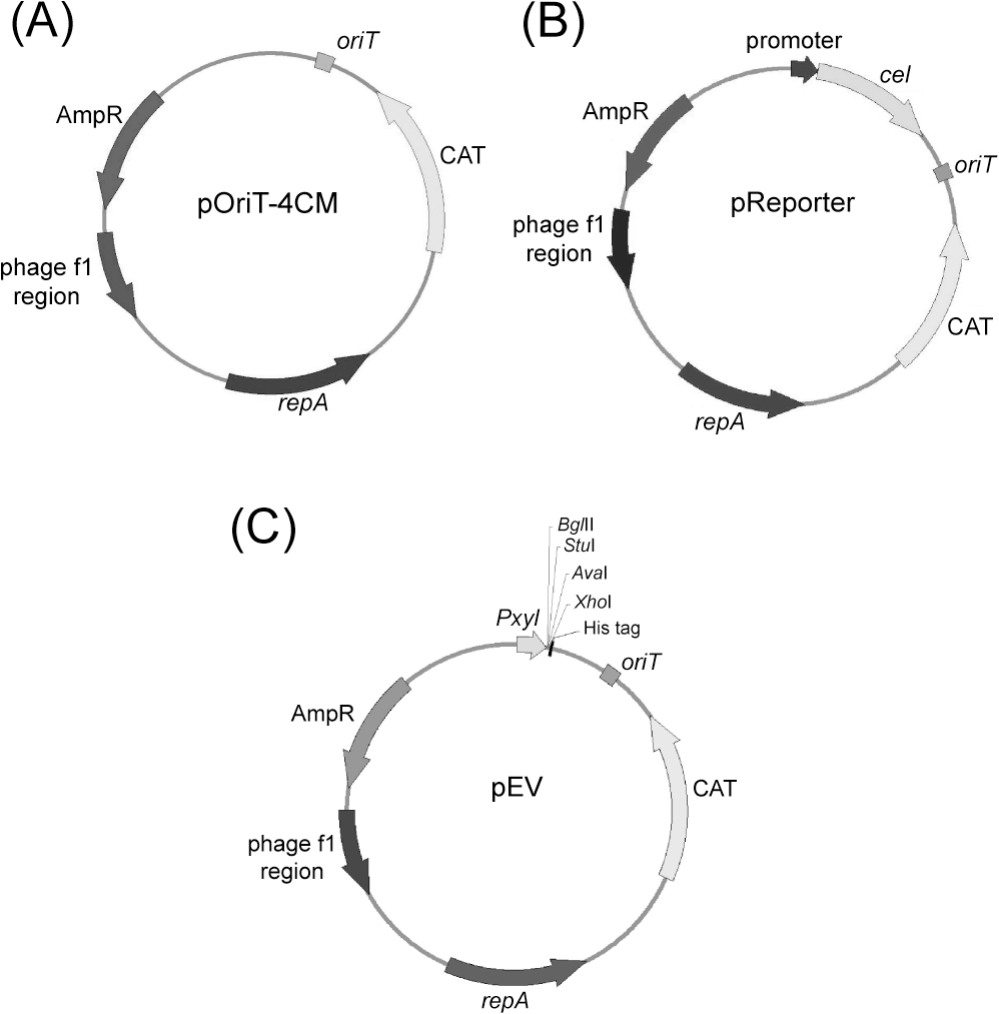
Schematic map of the shuttle vector pOriT-4CM (A), the reporter vector (B) and the expression vector pEV (C). *repA*, replication-related gene in *Pseudoalteromonas* sp. SM20429; *oriT*, origin of transfer; AmpR and CAT, selective marker for ampicillin resistance and chloramphenicol resistance, respectively; Phage f1 region, replication-related genes in *E*. *coli*; *cel308*, catalytic domain with its signal peptide of the cold-adapted cellulose gene; Pxyl, promoter of the xylanase gene from *Pseudoalteromonas* sp. SM20429.

The shuttle vector pOriT-4CM, capable of replicating in both *E*. *coli* and SM20429, was constructed based on the previously developed shuttle vector pWD2 [15]. As shown in Fig 1A, pOriT-4CM contains the origin of transfer (*oriT*) and the selective markers AmpR (for ampicillin resistance) and CAT (for chloramphenicol resistance). The phage f1 region on pOriT-4CM is responsible for plasmid replication in *E*. *coli*. Plasmid replication and segregational stability in SM20429 is attributed to *repA* and its flanking sequences (from plasmid pSM429) on vector pOriT-4CM [15].

pOriT-4CM was transferred into SM20429 by conjugational transfer using *E*. *coli* ET12567 (pUZ8002) as the donor. The donor cells and recipient cells were mixed and mated for 24 h. Then, the plates containing ampicillin and chloramphenicol were used for screening the transconjugants. In our previous study, we found that the donor *E*. *coli* ET12567 could not survive on the selective plate at 20°C after mating with *Pseudoalteromonas* stains for an unknown reason, though *E*. *coli* ET12567 harboring pOriT-4CM alone could grow on the selective plate at 20°C [20]. Therefore, the coloies grown on the screening plates were all transconjugant colonies. The transfer frequency was 4×10^−3^ transconjugants per donor cell, which provides a foundation for genetic manipulation in SM20429.

### Screening of a strong promoter for protein expression at low temperature

The putative promoters of genes encoding a cold shock protein (CspA), a heat shock protein (DnaK) and a xylanase were each cloned from BSi20429. Three promoter reporter vectors, p4xc (containing the promoter of the xylanase gene), p4cc (containing the *cspA* promoter) and p4dc (containing the *dnaK* promoter), were constructed based on the shuttle vector pOriT-4CM. Cold-adapted cellulase Cel308, from the Arctic sea ice strain *Pseudoalteromonas* sp. BSw20308, was used as the reporter (Fig 1B) [15].

**Fig 2:**
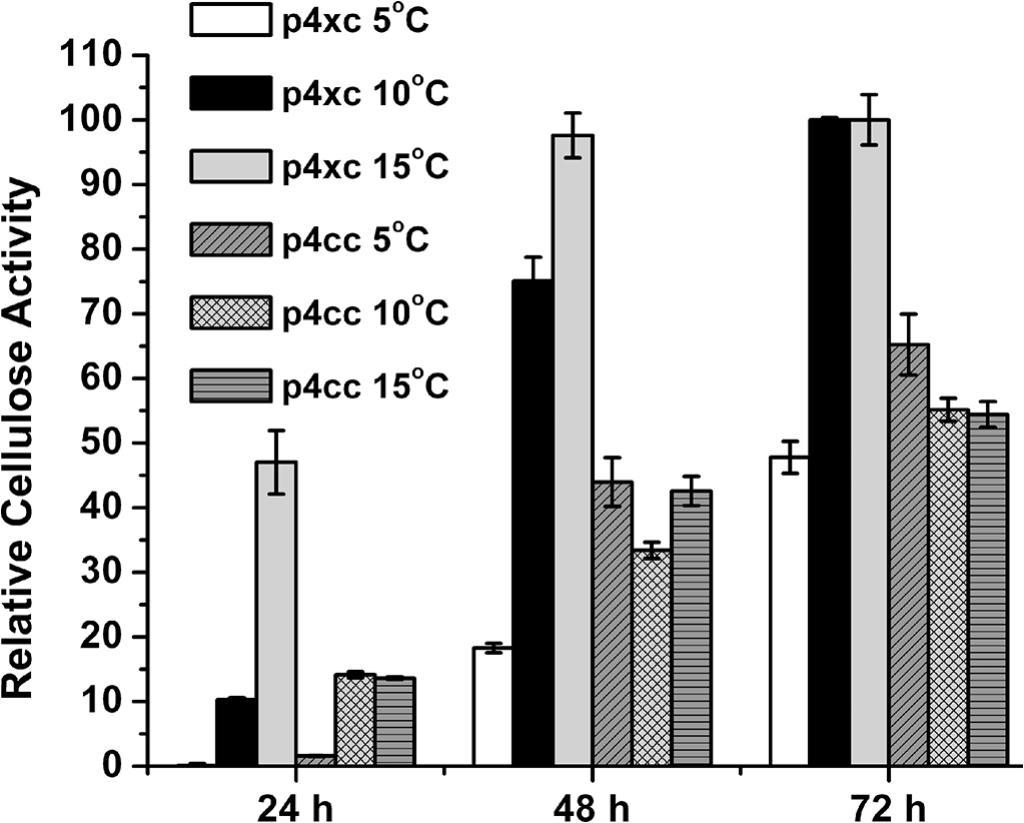
Cellulase activity of *Pseudoalteromonas* sp. SM20429 harboring promoter reporter vectors p4xc or p4cc at different temperatures. The activity of the cellulase expressed by p4xc at 15°C for 72 h was taken as 100%.

Each of the reporter vectors (p4xc, p4cc or p4dc) was transferred into SM20429 and cultured at 5°C, 10°C and 15°C. The extracellular cellulase activity was measured after 24 h, 48 h and 72 h of cultivation. No cellulase activity was detected in the culture of SM20429 harboring p4dc, suggesting that the promoter of *dnaK* was most likely not suitable for protein expression in SM20429. At both 10°C and 15°C, SM20429 harboring p4xc showed a much higher cellulase activity than the transformants containing p4cc (Fig 2), indicating that the promoter of the xylanase gene is more stronger than the *cspA* promoter at a low temperature. Therefore, the promoter of the xylanase gene was chosen for protein expression in SM20429 at a low temperature.

### Construction of the expression vector for protein expression and purification

To construct an expression vector convenient for gene cloning and protein purification, a DNA fragment containing the xylanase gene promoter from BSi20429, multiple cloning sites and a His tag was introduced into the shuttle vector pOriT-4CM. The resultant vector, pEV, was used as an expression vector in SM20429, which contained the cold-adapted promoter, multiple cloning sites and the His tag (Fig 1C).

### Pseudoalterin expression at a low temperature and purification

**Fig 3:**
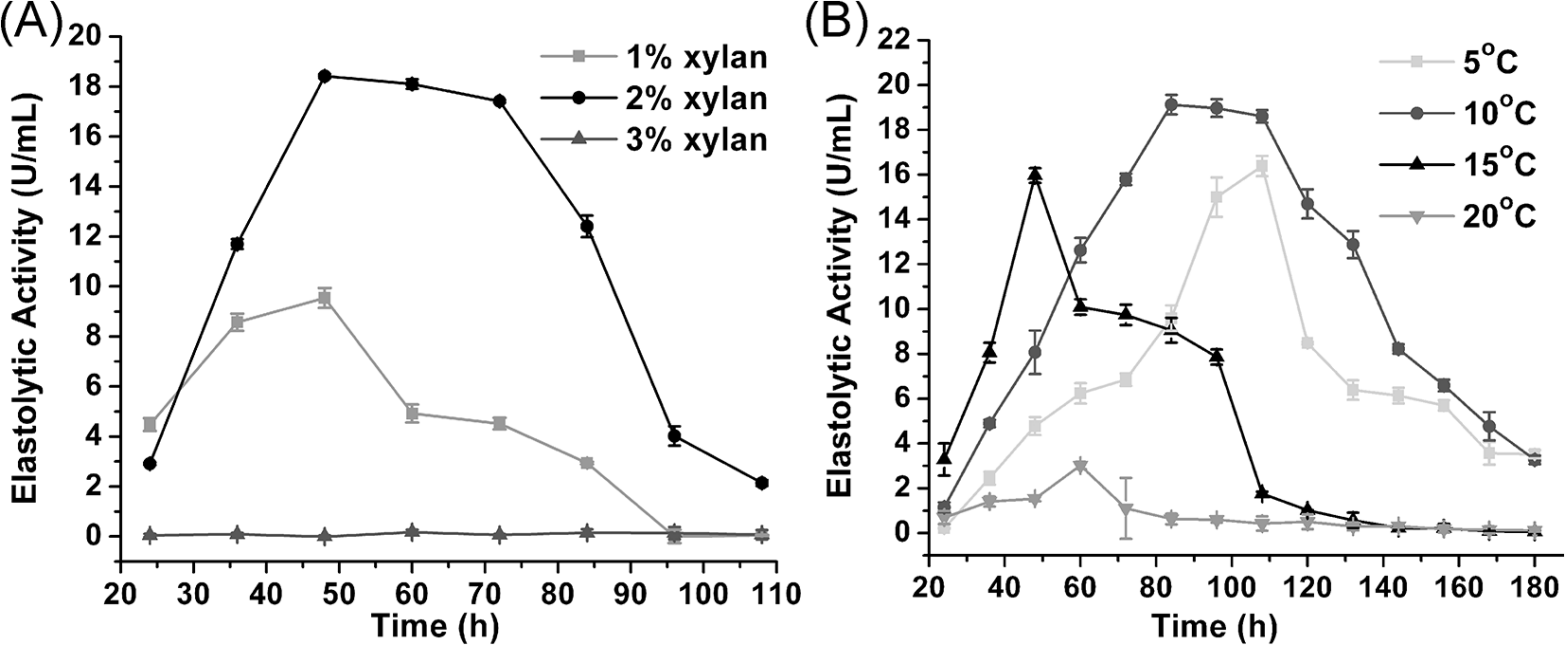
Pseudoalterin production after induction under different conditions. (A) *Pseudoalteromonas* sp. SM20429 harboring pEV-Psn was induced by different concentrations of oat spelt xylan at 15°C. (B) *Pseudoalteromonas* sp. SM20429 harboring pEV-Psn was induced by 2% oat spelt xylan at different temperatures.

**Fig 4:**
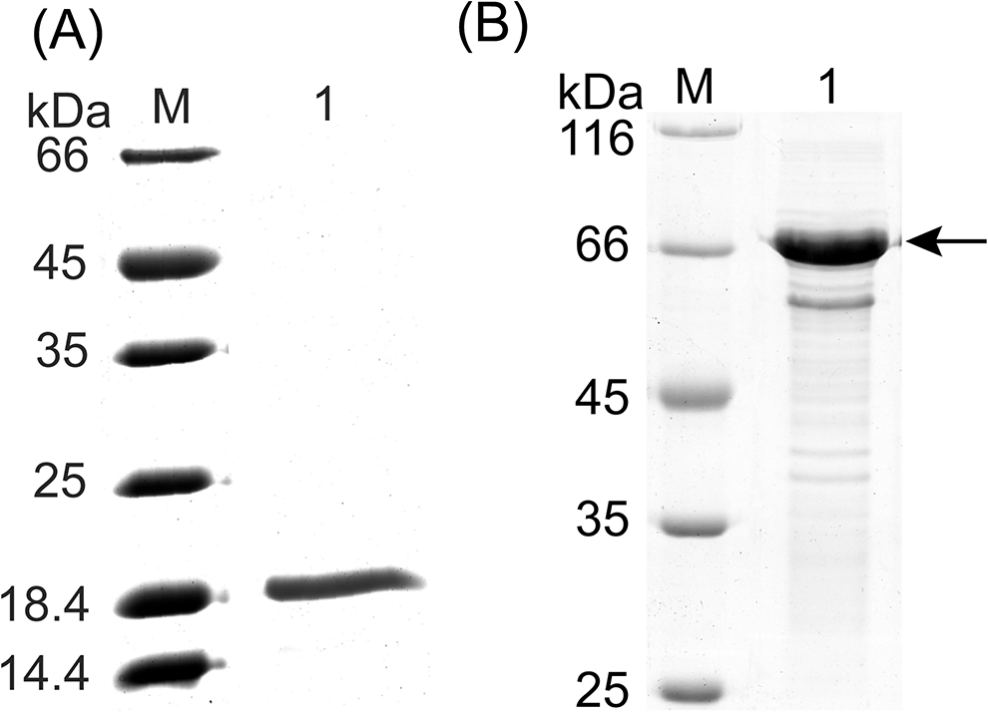
Pseudoalterin expressed by SM20429 and by *E*. *coli*. (A) SDS-PAGE analysis of the purified pseudoalterin from SM20429. M, marker; lane 1, the purified pseudoalterin. (B) SDS-PAGE analysis of pseudoalterin expressed by *E*. *coli* BL21 (DE3) after cloning the pseudoalterin gene into the vector **pGEX-4T-1** and induced by IPTG at 15°C. M, marker; lane 1, soluble recombinant pseudoalterin fused with a GST tag.

Vector pEV-Psn, containing the pseudoalterin gene, was introduced into SM20429 by conjugation. Using oat spelt xylan as an inducer, pseudoalterin was successfully expressed in its mature form by SM20429. After optimization of the induction conditions, an expression level of 15–20 U/ml was detected in the SM20429 culture with induction conditions of 2% oat spelt xylan at 10–15°C for approximately 48 h (Fig 3). The recombinant pseudoalterin was purified from the culture by Ni affinity chromatography (Fig 4A), indicating that the recombinant pseudoalterin contained a His tag, as expected. The yield of the recombinant protein was 1.2 mg/l culture. The recombinant pseudoalterin showed a molecular mass of approximately 19 kDa, equal to that of the native enzyme [11]. N-terminal amino acid sequencing showed that the recombinant pseudoalterin had the same N-terminal sequence (ATFTMNLPWS) as the native enzyme [11]. Substrate specificities toward proteins were compared between recombinant and native pseudoalterin. As shown in Table 3, recombinant and native pseudoalterin displayed similar substrate specificities. These results indicate that the recombinant pseudoalterin could reach its proper mature form in SM20429. In contrast, when recombinant pseudoalterin was expressed with a His tag in *E*. *coli*, it existed as an insoluble inclusion body (data not shown). When the recombinant pseudoalterin was expressed with a GST tag in *E*. *coli*, the recombinant pseudoalterin existed as a soluble precursor form, displaying a protein band of approximate 70 kDa, fused with the GST tag (Fig 4B). Therefore, pseudoalterin is a cold-adapted enzyme that has difficulty reaching its mature form using the *E*. *coli* expression system.

**Table 3:** Comparison of the substrate specificity between recombinant pseudoalterin and native pseudoalterin.

Substrate	Activity of recombinant pseudoalterin (units/mg)	Activity of native pseudoalterin (units/mg)
Casein	0	0
Elastinorcein	233.5 ± 0.6	292.8 ± 4.7
Gelatin	8.6 ± 1.9	5.5 ± 1.0
Bovine-insoluble type I collagen fiber	9.4 ± 0.1	4.3 ± 0.5

### Expression and purification of other cold-adapted proteins

**Fig 5:**
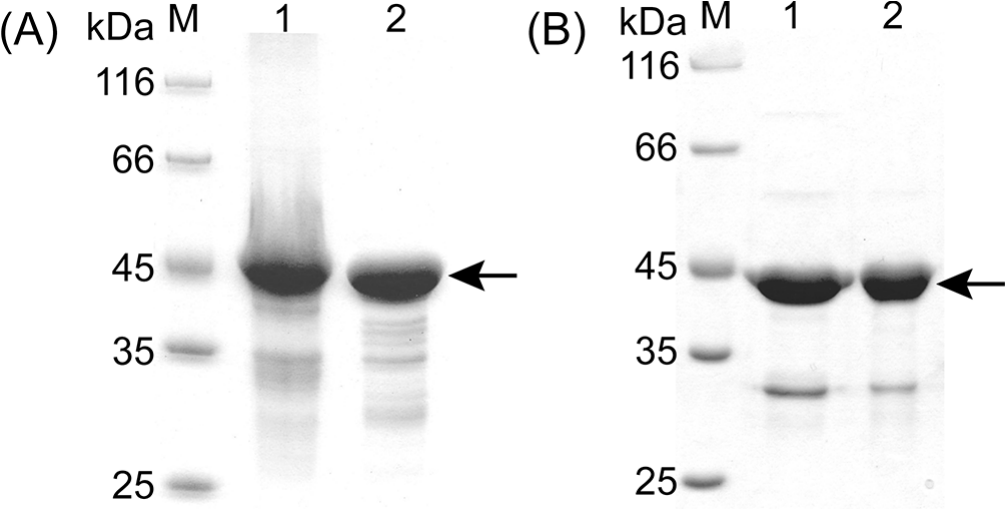
UDP-GlcNAc 2-epimerase and UDP-ManNAc dehydrogenase expressed by SM20429 and *E*. *coli*. (A) SDS-PAGE analysis of the purified proteins expressed by *E*. *coli* BL21 (DE3) M, marker; lane 1, UDP-ManNAc dehydrogenase; lane 2, UDP-GlcNAc 2-epimerase. (B) SDS-PAGE analysis of the purified proteins expressed by SM20429. M, marker; lane 1, UDP-GlcNAc 2-epimerase; lane 2, UDP-ManNAc dehydrogenase.

To further explore the applicability of the constructed expression system, two additional cold-adapted proteins, UDP-N-acetylglucosamine 2-epimerase (UDP-GlcNAc 2-epimerase) and UDP-N-acetyl-D-mannosamine dehydrogenase (UDP-ManNAc dehydrogenase) from the deep sea psychrophilic bacterium *Pseudoalteromonas* sp. SM9913, were expressed in the constructed *Pseudoalteromonas* expression system and compared with those expressed in the *E*. *coli* system. UDP-GlcNAc 2-epimerase and UDP-ManNAc dehydrogenase catalyze two continuous reactions during the synthesis of the exopolysaccharide (EPS) [24, 25]. When they were expressed in the *E*. *coli* T7 promoter system, the recombinant proteins were both purified from the intracellular space of *E*. *coli* by Ni affinity chromatography. Recombinant UDP-GlcNAc 2-epimerase showed a molecular weight of 42 kDa and recombinant UDP-ManNAc dehydrogenase showed a molecular weight of 45 kDa (Fig 5A). To express these two proteins in SM20429, two expression vectors were constructed: pEV-UDP-E for UDP-GlcNAc 2-epimerase and pEV-UDP-D for UDP-ManNAc dehydrogenase. Both of the proteins were expressed in SM20429 at 15°C and were induced by 2% oat spelt xylan. The recombinant proteins were both purified from the intracellular space of SM20429 by Ni affinity chromatography, in the same fashion as those expressed in the *E*. *coli* system. Using SDS-PAGE analysis, the molecular weights of the recombinant UDP-GlcNAc 2-epimerase and UDP-ManNAc dehydrogenase expressed in SM20429 were the same as the weights of those expressed in *E*. *coli* (Fig 5B). However, the expression efficiency of these two proteins in SM20429 (≈ 3 mg/g cells) was much lower than in *E*. *coli* (≈ 16 mg/g cells), suggesting that the expression efficiency of the cold-adapted *Pseudoalteromonas* expression system still needs further improvement.

## Discussion

Recombinant protein expression systems play an important role in the structural and functional analysis of proteins and their applications. To date, many expression systems have been developed using various organisms as hosts, including bacteria [26], eukaryotic microorganisms [27], insects [28], and mammalian cells [29]. However, there are still many proteins that are difficult to express via a recombinant protein expression system due to their low thermostability, toxicity to the host cell or tendency to form inclusion bodies. Therefore, alternative expression systems are still necessary to study these proteins. To express cold-adapted proteins, Ferrer et al. expressed the chaperones Cpn60 and Cpn10 of a psychrophilic bacterium in *E*. *coli* so that *E*. *coli* could grow at a low temperature[30, 31]. They successfully expressed a temperature-sensitive esterase in the modified cold-adapted *E*. *coli* [32]. Their work emphasizes the importance of expressing cold-adapted proteins at a low temperature using cold-adapted strains. Although the modified cold-adapted *E*. *coli* can grow at a low temperature to facilitate cold-adapted protein expression, it may still not be suitable for expressing proteins that cannot reach their mature form via autoprocessing, owing to the lack of some enzymes in *E*. *coli* that are required for processing protein precursors.

In the previous work, we attempted to develop a system for cold-adapted protein expression. An expression vector pWD2 harboring the *Plac* promoter from *E*. *coli* was constructed based on the cryptic plasmid pSM429 from the sea-ice bacterium *Pseudoalteromonas* BSi20429; however, proteins driven by the *Plac* promoter can only be expressed at 25–30°C [15]. Therefore, we attempted to further improve this expression system in this study. In the previous study, vector pWD2 was transferred into host SM20429 by electroporation [15]. In the present study, the shuttle vector pOriT-4CM was constructed based on the pWD2 model and a conjugal transfer system was developed. To find a strong promoter at low temperatures, thereby replacing the *Plac* promoter, several promoter reporter vectors were constructed based on the pOriT-4CM vector. The promoter of the xylanase gene from BSi20429 showed a good ability to express protein at low temperatures. Based on these results, the heterologous expression vector pEV was constructed, containing the promoter of the xylanase gene, multiple cloning sites and a His tag, thereby resulting in a convenient system for gene cloning and protein purification.

With SM20429 as the host and pEV as the expression vector, we attempted to express *Pseudoalteromonas* enzymes that cannot be maturely expressed in *E*. *coli* and cold-adapted proteins. Pseudoalterin, which is secreted by *Pseudoalteromonas* sp. CF6-2 from deep sea sediment, is a cold-adapted M23 metalloprotease that cannot be maturely expressed in *E*. *coli* [11]. Using the SM20429 expression system and induced by xylan, pseudoalterin was expressed as an extracellular active enzyme at 10–15°C. It was then purified by affinity chromatography, which showed the same N-terminal sequence and similar substrate specificity as the native pseudoalterin. In contrast, pseudoalterin could not be expressed as a soluble protein when expressed with a His tag in *E*. *coli*, and it could only be expressed into its precursor form when expressed with a GST tag in *E*. *coli*. Therefore, the expression system we constructed may be able to express *Pseudoalteromonas* enzymes that cannot be maturely expressed in *E*. *coli*.

Furthermore, we compared the expression of two cold-adapted proteins in SM20429 and in *E*. *coli*. The cold-adapted enzymes UDP-GlcNAc 2-epimerase and UDP-ManNAc dehydrogenase from *Pseudoalteromonas* sp. SM9913 were both successfully expressed by the *Pseudoalteromonas* expression system and the *E*. *coli* system. The recombinant proteins expressed in both systems were all intracellular soluble proteins and showed the same molecular weights. However, the *Pseudoalteromonas* expression system had much lower expression efficiency for these two proteins than the *E*. *coli* system, probably because the expression level of the xylanase gene promoter is weaker than the T7 promoter. Therefore, our results suggest that cold-adapted proteins can be expressed in the *Pseudoalteromonas* expression system, though may with a low expression efficiency.

Tutino et al. has developed a cold-adpated expression system in *Pseudoalteromonas haloplanktis* TAC125 [12, 13], and several proteins that can be maturely expressed in *E*. *coli* [33–36] have been expressed by this system. However, it is still unclear whether proteins intractable for the *E*. *coli* system can be expressed by this system. In addition, the expression vector in this expression system does not have a His tag, which would make it inconvenient to purify the recombinant proteins. In this study, using a promoter that is strong at a low temperature and a psychrophilic bacterial strain, *Pseudoalteromonas* sp. SM20429, we constructed a cold-adapted *Pseudoalteromonas* expression system that can express proteins at 10–15°C, with xylan as an inducer. In addition, multiple cloning sites and a His tag was constructed in the expression vector, which is convenient for protein expression and purification. Our results show that the *Pseudoalteromonas* expression system we developed is useful for expressing *Pseudoalteromonas* enzymes that cannot be maturely expressed in *E*. *coli*, such as pseudoalterin, which is helpful for the further study of such enzymes. Our results also support the future application of this cold-adapted expression system for the expression of cold-adapted proteins, although it still needs further improvement. Therefore, the cold-adapted *Pseudoalteromonas* expression system that we developed will provide an alternative choice for protein expression, especially for the *Pseudoalteromonas* proteins that are intractable for the *E*. *coli* system.
